# Remote Sensing of the Water Storage Dynamics of Large Lakes and Reservoirs in the Yangtze River Basin from 2000 to 2014

**DOI:** 10.1038/srep36405

**Published:** 2016-11-04

**Authors:** Xiaobin Cai, Lian Feng, Xuejiao Hou, Xiaoling Chen

**Affiliations:** 1 Institute of Geodesy and Geophysics, Chinese Academy of Sciences, Wuhan, 430077, China; 2 Key Laboratory of Monitoring and Estimate for Environment and Disaster of Hubei, Wuhan, 430077, China; 3 State Key Laboratory of Geodesy and Earth’s Dynamics, Wuhan, 430077, China; 4 State Key Laboratory of Information Engineering in Surveying, Mapping and Remote Sensing, Wuhan University, Wuhan 430079, China

## Abstract

Basin-scale water volumes of lakes and reservoirs are difficult to obtain due to a number of challenges. In this study, area-based water storage estimation models are proposed for large lakes and reservoirs in the Yangtze River Basin (YRB). The models are subsequently applied to Moderate Resolution Imaging Spectroradiometer (MODIS) observations of 128 large lakes and 108 reservoirs between 2000 and 2014, and the first comprehensive map of the temporal and spatial dynamics of water storage in large water bodies in the YRB is provided. The results show that 53.91% of the lakes experienced significant decreasing trends in water storage during this period, and the total water storage in lakes showed a decreasing trend of 14 million m^3^ month^−1^. By contrast, a monthly mean increase of 177 million m^3^ was observed for water storage in reservoirs. Our analysis revealed that the pronounced increase in reservoirs was primarily due to the rapid water level increase in the Three Gorges Reservoir in recent years, while understanding the water loss in lakes requires additional studies. The long-term data presented in this study provide critical baseline information for future water resource monitoring and regulation in the YRB and China.

Water in lakes and reservoirs is one of the most important components in the terrestrial water system^1^,^2^. Accurate and timely information regarding the water resources in lakes and reservoirs, in terms of both inundation area and water storage, is essential for the effective management of competing uses, such as flood control, drought mitigation, agricultural irrigation, recreation, and fisheries^3^,^4^,^5^. This is particularly true in the Yangtze River Basin (YRB), with >1000 (number) or >10,000 km^2^ of lakes and reservoirs of various sizes. The water resources in the basin are one of the most concentrated distributions of freshwater in Asia or even the world, providing water supplies to more than 50 million people in the basin^6^,^7^.

Due to its ecological and social importance, considerable efforts have been made to monitor the surface water dynamics in the YRB. For example, the inundation areas of certain individual lakes in the YRB have been quantified using decades of remote sensing observations. These lakes include Poyang Lake^8^,^9^, Dongting Lake^9^,^10^,^11^,^12^, and Taihu Lake^13^. Comprehensive studies of hydrologic areas such as the Jianghan Plain and the middle and downstream reaches of the YRB have been conducted at the sub-basin scale using long-term satellite images^6^,^14^,^15^,^16^. In general, it is relatively easy to delineate inundation areas using optical remote sensing data, as the water signal is much lower than the land signal, especially in the NIR spectrum due to significant water absorption^17^.

Unfortunately, the water storage of natural lakes or man-made reservoirs in the YRB has rarely been studied, as it is difficult to characterize using traditional field surveys or remote sensing methods. Theoretically, the estimation of the water volume of a lake or reservoir requires both bottom topography and water level (or water surface elevation), where the water storage is the integration of the difference between the water level and the bottom. Water levels can be determined using gauged hydrological stations, but this is difficult at large scales and in less developed regions where hydrological stations are not available^18^. Satellite radar altimetry provides a complementary means of obtaining water surface elevations^19^. However, the sparsely distributed data constrain the large-scale application of this technique. With synoptic and frequent observations, optical remotely sensed images are able to delineate water/land the boundaries, where the water surface elevations can be determined based on their overlap with boundaries and the bottom typography^20^. Conversely, determining the bathymetry of a lake or reservoir tends to be more challenging, requiring special equipment and considerable labour and money^21^. Thus, the bottom topographical measurements of hundreds of large water bodies in the YRB appear to be practically impossible.

As the surface area of a lake or reservoir is often highly correlated with water volume, global and regional empirical relationships have been proposed to estimate water storage using only inundation area^22^,^23^. Area-based water volume calculation models in the YRB have also been proposed by Yang and Lu^7^ for both lakes and reservoirs, where Landsat-derived surface areas and the officially reported storage capacities were used to develop area-based water volume estimation models for different inundation levels. However, due to significant seasonal and inter-annual variability in the surface areas in the Middle and Lower Yangtze Basin (MLYB), the water areas delineated using one Landsat TM/ETM+ image may not accurately represent the storage capacity of a lake or reservoir. In addition, the short- to long-term dynamics of the stored water resources in lakes and reservoirs in the YRB cannot be obtained using one period of observations.

Motivated by the urgent need for accurate information regarding basin-scale water storage in the YRB, this study focuses on the following goals: (a) recalibrate the empirical models to quantify the water volume of lakes and reservoirs using remotely sensed inundation areas, (b) evaluate the temporal and spatial dynamics of water storage in large lakes and reservoirs using long-term remote sensing observations, and (c) build a 15-year environmental data record (EDR) for large inland water bodies in the YRB that can serve as an important source of information for future water resource regulations in this region and in China.

## Data and Methods

### Selection of Lakes and Reservoirs

**Figure 1:**
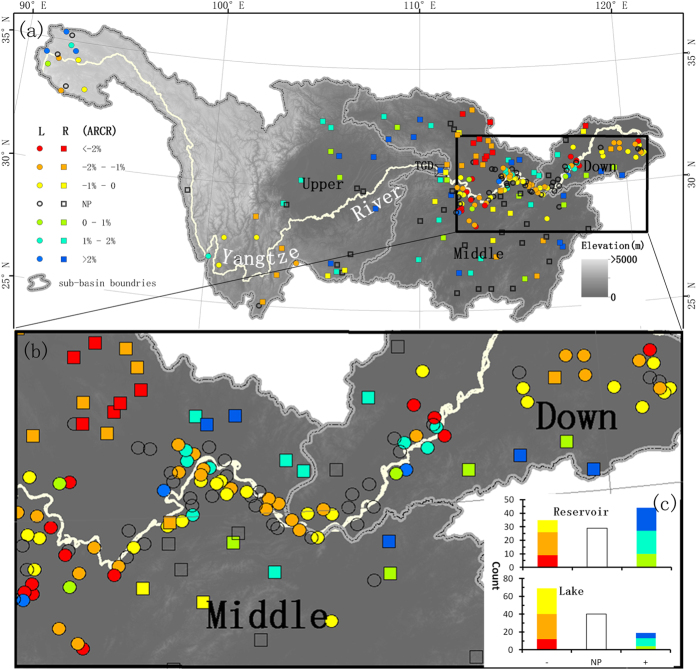
(**a**) The locations of the large lakes (circles) and reservoirs (squares) studied in the YRB. Different colours indicate the annual rates of change in water storage from 2000 to 2014. The details of the middle stream to downstream YRB are enlarged in (**b**). (**c**) The histograms show the number of lakes and reservoirs that have different long-term trends over the observed period. (The map was created using ESRI ArcGIS 10.1, http://www.esri.com/software/arcgis/arcgis-for-desktop).

Considering the relatively low spatial resolution of MODIS data, only lakes with surface areas of >8 km^2^ and reservoirs with capacities of >0.1 km^3^ were selected in this study. The areas and capacities of the lakes and reservoirs in the YRB were obtained from the official reports of several water conservancy agencies in China, such as the China Lake Scientific Database, Chinese lake catalogues^24^ and the water conservancy information system from the Development and Research Center of the Ministry of Water Resources. The locations of the 128 (in number) selected lakes and 108 (in number) selected reservoirs are shown in Fig. 1.

### Delineation of Inundation Areas

MODIS 8-day surface reflectance data composites with spatial resolutions of 250 m (MOD09Q1 and MYD09Q1) and 500 m (MOD09A1 and MYD09A1) were used in this study to determine the inundation areas of the selected lakes and reservoirs. A total of 2516 composites were downloaded from the NASA Land Processes Distribution Active Archive Center (https://ladsweb.nascom.nasa.gov/), including Terra data from 2000 to 2014 and Aqua data from 2002 to 2014. The 500 m data were re-sampled to 250 m using a sharpening method^25^. The MODIS 8-day composite products represent the best possible observations during an 8-day period, as they are characterized by high observation coverage, low viewing angle, absence of clouds, etc.

A region of interest (ROI) for each lake and reservoir was determined based on a wet season MODIS image, and the size of the ROI for each water body was larger than the maximum surface area. High-resolution images from Google Earth were used to define the boundaries of some reservoirs that were constructed on rivers (such as the Three Gorges Reservoir, or TGR) or built in the rugged and mountainous upper Yangtze reaches. Similar to that used by Wang *et al*.^6^, an interactive graphical user interface (GUI) was developed to classify water and land using human intervention. MODIS Red-Green-Blue (R: 645, G: 555, B: 469) composites and the normalized difference water index (NDWI)^17^ of each ROI were loaded into the GUI. The NDWI threshold was then changed manually until the corresponding water/land interface agreed well with high-contrast pixels in both the RGB and NDWI images. Note that due to the limited spatial resolution of MODIS imagery, lakes and reservoirs with very narrow surface inundations were excluded from this study, eliminating large errors associated with mixed pixels. In addition, ROIs with residual clouds were excluded when determining the thresholds. Selected high-resolution (30-m) images collected by the Landsat-8 OLI were obtained from the US Geological Survey (http://landsat.usgs.gov/), and the inundation areas classified from these high-resolution images were used to validate the results of the concurrent lower-resolution MODIS observations.

### Area-Based Water Storage Estimation Model

Previous studies have demonstrated that the storage capacities of reservoirs are highly correlated with surface area at the regional and global scales^23^,^26^, and the relationship is also significant for natural lakes. Thus, the water storage in a water body can be estimated using its surface area, and the model can be expressed as follows:


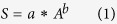


where S is the estimated storage and A is the area of the water body. Both a and b are constants.

The maximum inundation area of a water body between 2000 and 2014 was assumed to be associated with the storage capacity, as it is likely to hold the maximum water storage. Note that the maximum water areas of different water bodies differed due to varied local meteorological and hydrological conditions that regulate inundation. Using maximum area-storage capacity pairs from 128 lakes and 108 reservoirs, the constants a and b in Eq. (1) were calibrated separately for lakes and reservoirs, and from which the new area-based storage models can be developed.

### Trend Analysis

With MODIS-delineated water surface areas from 2000 to 2014 and the proposed area-based storage capacity models, the water storages of the selected lakes and reservoirs at any time in this period can be estimated. The 15-year water storage information makes it possible to reveal the long-term dynamics of the surface waters in the YRB. In practice, the total monthly storages of all the selected lakes (S_l_), reservoirs (S_r_) and total surface waters (S_l+r_ = S_l_ + S_r_) were derived, and the annual mean values were calculated as the mean values of the 12 months in a year. To reveal the long-term trends during the observed period, S_l_, S_r_, and S_l+r_ were decomposed into two components: (1) a seasonal term that includes the monthly climatologies between 2000 and 2014, and (2) the monthly anomalies, estimated as the differences between the monthly water storages and the monthly climatologies, which were denoted as S_a,l_, S_a,r_, and S_a,l+r_ for lakes, reservoirs and the total storages, respectively. Then, a linear regression over the long-term anomalies was used to determine whether a statistically significant increasing or decreasing trend existed for S_l_, S_r_, and S_l+r_. Meanwhile, the Breaks For Additive Season and Trend (BFAST) approach was also introduced to find the possible breakpoints when abnormal changes occurred in the water storages^27^. The same decomposition and linear regression methods were also applied to individual lakes or reservoirs to obtain the long-term trend of each water body.

### Analysis of the Meteorological Measurements

Monthly precipitation data between 2000 and 2014 were obtained from NASA’s Tropical Rainfall Measuring Mission monthly product (TRMM 3B43, V6)^28^. Concurrently, the MODIS Global Terrestrial Evapotranspiration (ET) Data Set (MOD16)^29^ was obtained from the Numerical Terra dynamic Simulation Group (NTSG) (http://ntsg.umt.edu/project/mod16). The precipitation and ET data were integrated over the entire YRB and were used to examine the long-term changes in the meteorological conditions in this region.

## Results

**Figure 2:**
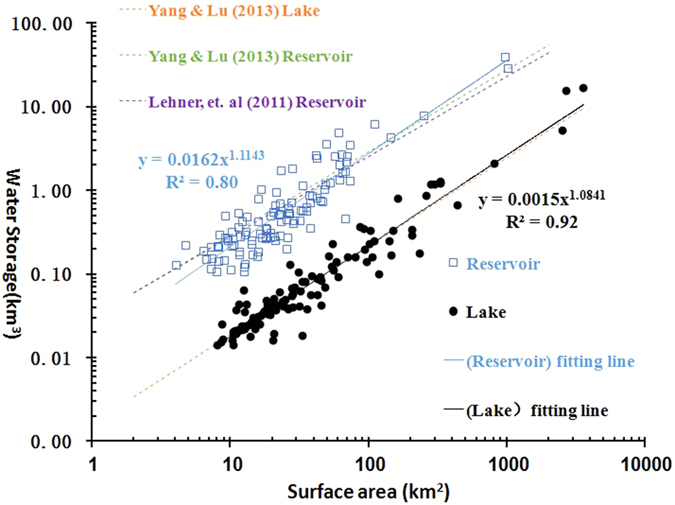
Relationships between maximum surface areas between 2000 and 2014 and the officially reported capacities for lakes (n = 118) and reservoirs (n = 108). The relationships proposed in previous studies are also plotted.

Significant power relationships were found between the water areas and storages of both lakes and reservoirs in the YRB (Fig. 2). With three to four orders of dynamic ranges for surface areas and water storages, the points plotted near the regression lines, and the coefficient of determination (R^2^) reached 0.92 and 0.80 for lakes and reservoirs, respectively. The root mean square differences (RMSD) between the estimated regression and reported capacities were 15.25% and 33.68% for lakes and reservoirs, respectively. The power regression model of reservoirs is larger than that of lakes by ~1 order of magnitude, suggesting that the storage of reservoirs would be much larger than that of lakes with the same water area. Although the models were similar to those used in previous studies^7^,^22^,^30^, differences can be observed for both lakes and reservoirs. Specifically, the reservoir storage predicted using the current model was slightly higher than storage predicted by Yang and Lu^7^ and Lehner^22^ for relatively large surface areas (>100 km^2^), while the opposite is true when the inundation area is small (<100 km^2^). Conversely, the lake storage calculated using the current model is slightly larger than that calculated by Yang and Lu^7^. Note that Yang and Lu^7^ used more water bodies (185 lakes and 118 reservoirs)^7^ to establish their regression models because the Landsat measurements used in their study could be used to delineate smaller water bodies.

The variational patterns of all the lakes and reservoirs are illustrated in Fig. 1, where the annual rate of change over the observed period for each body is colour coded. Numerically, more than half (53.91% and 69 in number) of the selected lakes showed statistically significant decreasing trends (p < 0.05) in their water storages between 2000 and 2014. These lakes were primarily located in the MLYB and along the main stream of the Yangtze River. Meanwhile, only 14.84% of the lakes displayed increasing trends, and many of them are primarily located in the upper Yangtze River reaches, which is consistent with the findings of some previous studies^31^,^32^,^33^. The water volume increases in these lakes were mainly attributed to global warming-triggered glacier retreat in high mountains^32^,^33^. No apparent trends were found for the most of remaining lakes. Conversely, reservoirs were mainly located in the middle and upper YRB and are far away from the main stream, and the number of reservoirs with significant increasing trends (40.74%) was comparable to the number with significant decreasing trends (32.41%). However, increases primarily occurred in relatively large reservoirs where the mean water storage capacity was 2.1 km^3^, while the mean storage capacity was 0.4 km^3^ for those with decreasing trends. Similar increasing trends for reservoirs and decreasing patterns for lakes were also noted between 2000 and 2011 by Wang *et al*.^6^, who mainly focused on the inundation areas of large water bodies in the middle and lower Yangtze River Basin.

**Figure 3:**
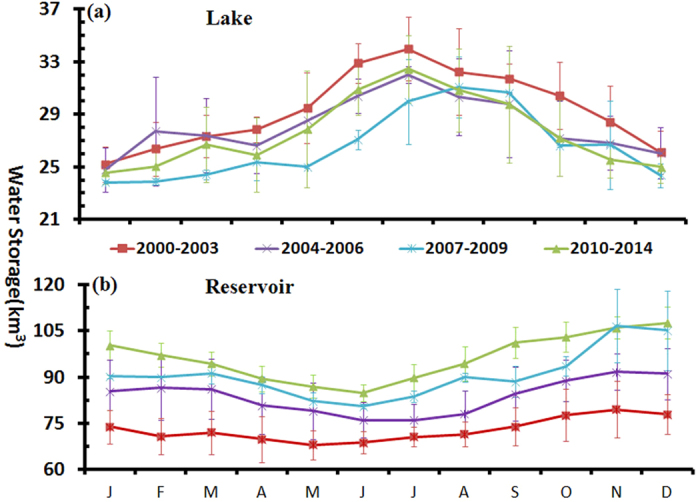
Monthly climatologies of the water storages of lakes (**a**) and reservoirs (**b**) during different periods, which are separated by BFAST-revealed breakpoints in 2003, 2006 and 2009.

The monthly S_l_ values of the 128 selected lakes ranged from 21.82 km^3^ (minimum) in May 2011 to 36.84 km^3^ in August 2002. Prominent seasonality exists in addition to the significant inter-annual variability, and the water storage in the wet season (summer months) reached more than twice that in the dry season (winter months) (see Fig. 3a). By contrast, intra-annual changes in S_r_ were less evident. Only small differences were found between the 12-month climatological water storages. S_r_ appeared to be much larger than S_l_ during the entire period, but especially during the latter half of the period when the annual mean S_r_ increased dramatically. The patterns of inundation area change were similar to those of water storage, as the former were estimated based on the latter. The maxima between 2000 and 2014 were 14251.55 km^2^ for lakes and 4076.24 km^2^ for reservoirs, respectively (see supplementary Fig. S1).

**Figure 4:**
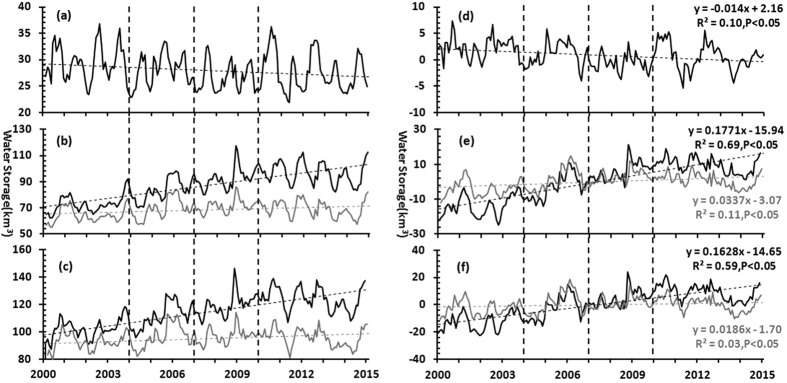
Long-term trends in the water storages of large lakes and reservoirs in the YRB from 2000 to 2014. The monthly water storages of lakes (S_l_), reservoirs (S_r_) and the total surface waters (S_l+r_ = S_l_ + S_r_) are demonstrated in (**a–c**), respectively. (**d–f**) Are the monthly anomalies of lakes, reservoirs and total storage (denoted as S_a,l_, S_a,r_, and S_a,l+r_ in the text), which were estimated as the differences between the monthly water storages and the corresponding monthly climatologies. In (**b,c,e,f**), the grey lines show the results without the Three Gorge Reservoir.

The long-term seasonal anomalies of lakes, reservoirs and total surface waters are plotted in Fig. 4d–f, which shows the inter-annual trends between S_a,l_, S_a,r,_ and S_a,l+r_. Specifically, S_a,l_ exhibited a decreasing trend of 14.00 million m^3^/month from 2000 to 2014, which is statistically significant (e.g., p < 0.05, R^2^ = 0.10). Conversely, a very pronounced trend can be found for S_a,r_ (p < 0.05, R^2^ = 0.69), which exhibited a monthly mean increasing rate of 177.10 million m^3^. Additionally, the monthly anomaly of the total surface waters (S_a,l+r_) also displayed an increasing trend of 162.80 million m^3^/month over the 15-year period, which is due to the dominant roles of reservoirs in the total surface water storage.

The annual mean water storages of lakes ranged between 25.29 km^3^ in 2011 and 30.37 km^3^ in 2010 (Table 1). Although seasonal anomalies (S_a,l_) showed a significant decreasing trend from 2000 to 2014, no obvious trend in S_l_ was found at the annual scale. By contrast, with a magnitude of 2–4 times that of S_l_, the annual mean S_r_ increased rapidly from <80 km^3^ before 2003 to >85 km^3^ after 2005, peaking at 98.93 km^3^ in 2010. Correspondingly, the total surface water storage (S_l+r_) increased from ~100 km^3^ in the first few years to ~120 km^3^ in the latter years of the 15-year period.

BFAST revealed three breakpoints in the trend components of both S_l_ and S_r_, which were in the years of 2002–2003, 2005–2006 and 2009–2010 (see Supplementary Fig. S2). With these breakpoints, the time series of water storage can be divided into four periods of 2000–2003, 2004–2006, 2007–2009 and 2010–2014. Sudden decreases were observed in the trend components of S_l_ at the breakpoints in 2003 and 2006, after which the trend remained relatively stable, although subtle increases can be found in the second period. By contrast, the trend components of S_r_ increased dramatically from the first three periods to the fourth period, and the values then decreased slowly during the last period. In addition to the significant inter-annual variabilities in the trend components, distinguishable differences can also be found between the monthly mean S_r_ values in the first two periods and the latter two periods. This difference was statistically significant in the majority of the 12 months (i.e., >2 standard deviations above “normal”). The monthly mean S_r_ increased from 72.86 km^3^ before 2004 to 83.68 km^3^ from 2004–2006, and it then increased to 96.30 km^3^ in the latter years, clearly indicating two regime shifts in reservoir storage. However, no similar patterns were found for the monthly mean values of S_l_.

## Discussion

### Accuracy Analysis

Ideally, the water storages of lakes and reservoirs should be calculated using their bathymetries. However, it is practically impossible to obtain the lake bottom topographies for hundreds of water bodies in the YRB, prohibiting error-free estimates of the water volumes. As a compromise, the area-based power relationships and MODIS-delineated inundation areas were proposed to derive the water storages of lakes and reservoirs in this study. Due to the relatively low spatial resolution of MODIS, the mixed pixels between the land/water interfaces can lead to potential uncertainties in the long-term dynamics of surface water storage. This problem can be solved with high-resolution remote sensing data (such as 30-m-resolution Landsat). However, the infrequent observations (16 days) and high probability of cloud coverage in the YRB make it difficult to obtain the maximum inundation area that corresponding to the storage capacity of each water body using Landsat, prohibiting the establishment of the optimal power relationship in Eq. (1). Additionally, the significant seasonal and inter-annual variabilities in inundation areas and water storages cannot be captured using infrequent Landsat observations. With a short revisiting period and moderate spatial resolution, MODIS appears to be the most suitable data source for the current study. Additionally, although it is difficult for MODIS data to map small water bodies due to its moderate spatial resolution (250 m), the exclusion of these small lakes and reservoirs would not influence the general trends in total water storage in the study area, as their contributions to the water storage in the YRB are limited.

Nevertheless, surface water areas derived using sporadic concurrent Landsat 8 OLI measurements with a 30-m resolution were used to validate the use of 250-m MODIS data (see Supplementary Fig. S3). The results of two independent observations exhibited good agreement (R^2^~1), with an RMSD of 20.27% (n = 62) for the inundation areas of lakes corresponding to an RMSD of 21.82% for the water storage estimation. Likewise, the RMSDs of the inundation areas and water storages of reservoirs were 22.55% and 23.38%, respectively (n = 78). Considering the uncertainties in the satellite-derived inundation areas and that the established power relationships are statistically independent, the uncertainties in the model-estimated water storages were 26.62% (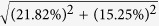) and 41.00% (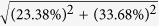) for each individual lake and reservoir, respectively. However, the uncertainties should be much smaller for large water bodies due to the relatively small impacts of mixed pixels on their water surface areas. For example, the two largest lakes in the YRB, Poyang Lake and Dongting Lake, account for 55.91% of the total storage capacity of all lakes, where the differences between MODIS- and Landsat-delineated inundation areas were 10.31% and 11.53%, respectively. The total water storage is mainly associated with large water bodies whose uncertainties should be much less than the mean levels of individual lakes or reservoirs. Notably, the water area delineated using any MODIS 8-day composite could represent the inundation conditions during the associated 8-day period, while the inundation conditions from Landsat-8 OLI data were obtained for a single day. Thus, the uncertainties in the MODIS-derived surface area involved the potential temporal discrepancies in the inundation conditions within an 8-day period. Additionally, the “true” inundation areas based on Landsat data also have some errors due to a number of reasons^34^; however, these errors were not considered in this study, as they were difficult to incorporate into the final water storage estimation.

Other consistency checks can also be used to verify the rationality of the proposed method for surface water storage estimation. First, the total water storage capacity of all the selected water bodies (206.9 km^3^) estimated using the power-based model (Eq. (1)) and the maximum inundation areas during the 15-year period was very similar to that of the officially reported water storage capacity (229.4 km^3^). Second, integrating the maximum inundation areas of large reservoirs and lakes in the YRB yielded a value of 18327.79 km^2^, approaching the high-resolution satellite data (CBERS CCD and Landsat TM/ETM)-derived result (19563.0 km^2^) of Ma *et al*.^35^. The larger value reported by Ma *et al*.^35^ was likely due to the inclusion of smaller lakes (1–10 km^2^). Third, the total maximum area of lakes (14251.55 km^2^) was consistent with the values estimated by Yang and Lu *et al*.^7^ using Landsat measurements (13366 km^2^). The difference between the two is likely because our result is the sum of the maximum area of each lake from MODIS images between 2000 and 2014, while the latter was acquired from one TM/ETM+ image in wet months between 2005 and 2008. Finally, the maximum inundation area (14251.55 km^2^) obtained here agrees well with that determined by Wang *et al*.^6^ based on daily 250-m and 500-m MODIS images (14828.9 km^2^). Differences between these values are likely because some of the gated or controlled lakes in the Yangtze Floodplain were considered lakes rather than reservoirs in their study.

The validity of the time series estimations of water storage can be further assessed using concurrently gauged water levels. Long-term monthly water level measurements in Poyang Lake and the TGR were obtained and compared to the corresponding MODIS-estimated water storages. The results show that both the seasonal and long-term patterns were consistent between the water level observations and water storages, with R^2^ values of >0.8 for both Poyang Lake and TGR, suggesting that the temporal variations in the water storages can be well represented using the remotely sensed estimates.

### Potential Driving Forces of the Water Storage Dynamics

The water storages of the lakes and reservoirs can be modulated by weather conditions in the YRB. For example, when the minimum annual mean precipitation was observed in 2011, the minimum value of S_l_ was also observed. By contrast, when the maximum precipitation occurred in 2010, the corresponding water storages in lakes and reservoirs were very large. However, when examining the long-term ET and precipitation data from 2000 to 2014 (see Supplementary Fig. S5), other than the noticeable seasonal cycles and inter-annual fluctuations, no significant trends can be found in these two components of the water budget in this region (see Supplementary Fig. S4). Thus, the roles of the two most important natural factors in significantly increasing the total surface water storage in the YRB are small.

The variations in S_r_ were likely associated with the impoundment of the Three Gorges Dam (TGD). The Three Gorges Reservoir (TGR) is the largest reservoir in the YRB in terms of water capacity (39.3 km^3^), and the two critical impounding times were associated with the first two BFAST-derived breakpoints (e.g., 2003 and 2006)^36^. The initial impoundment in 2003 raised the water level of the TGR from <70 m to 135 m, leading to a dramatic increase in the water volume in TGR and thus S_r_ (see Fig. 4e). Moreover, S_r_ reached to 89.70 km^3^ when the water level in the TGR increased to 155 m during the second impoundment in 2006. The MODIS-estimated results of S_r_ showed that the water storage of the TGR has increased by 25.93 km^3^ over the past 15 years, representing 81.82% of the increases in all large reservoirs in the YRB during this period. This reflects the dominant influence of the TGR on S_r_ in the entire YRB. However, the water volume in the TGR was regulated by the operation of the TGD for electricity generation, flood/drought mitigation and other purposes, leading to S_r_ fluctuations after the TGD was impounded. Note that when the water storage of the TGR was not included in the analysis, significant increasing trends remained for both S_a,r_ and S_a,l+r_, although the slope of the increase was flatter (see Fig. 4b,c), reflecting a general increase in the water storages of reservoirs in the YBR.

However, the cause of the moderate decline in lake water storage, especially in lakes in the middle and lower reaches of the YRB, remains unknown. Yet, there are several possible reasons. First, rapid increases in industrial growth and human water consumption in recent years may have led to the lake shrinkage^37^. This is likely the case in the downstream YRB, where the most intensive urbanization and industrialization has occurred in China^38^. Second, the changes in hydrological conditions due to operating the TGD generally reduced lake size, especially because the Yangtze River is connected to Poyang Lake and Dongting Lake. In this study, significant decreases in inundation areas were found for these two large freshwater lakes over the past decade^9^. Third, the decline in lake storage may be used to offset the rapid increase in reservoir storage based on the water mass balance in the YRB, as insignificant changes were found for precipitation, ET, and the annual water discharge of the Yangtze River^39^. Thus, the increase in water impoundment by reservoirs (such as the TGR) may reduce the volume of water discharged to the Yangtze floodplain, where most lakes with decreasing areas are located^6^. However, the exact reasons must be further investigated when more hydrological and meteorological data are available.

### Implications for Future Applications

Changes in the mass balance of the Earth are primarily associated with variations in terrestrial water storage (TWS), which consists of surface water, groundwater, soil water, snow and ice^40^. Currently, a typical approach to understanding large-scale water cycles is to disaggregate satellite signals from the Gravity Recovery and Climate Experiment (GRACE) into different components based on combinations of soil water and groundwater information obtained from hydrological models. However, the contributions of surface water in these hydrological models are ignored (such as the Global Land Data Assimilation System (GLDAS)) or simulated using a few global-based calibration parameters that may not be suitable in the YRB. Moreover, the water storage in reservoirs is generally modulated by human activities, making it difficult to simulate using hydrological models. Thus, the remotely sensed water mass dynamics of lakes and reservoirs analysed in this study would help to better determine the water budget of the TWS in the YRB.

Reservoirs are an important water component at the global scale, and this is especially true in China. By the end of 2010, China was ranked fourth in the world in terms of water storage capacity in reservoirs, accounting for 10% of the total reservoir capacity in the world^41^. The construction of man-made reservoirs has never stopped in China, and the expected capacity could reach 41.4 km^3^ based on four large cascade reservoirs (Wudongde, Baihetan, Xiluodu and Xiangjiaba) that are planned in the YRB^42^, representing >24% of the current levels. Timely information regarding water storage is crucial for developing effective measures to alleviate floods and droughts; however, this information is technically challenging in large regions with hundreds of reservoirs using traditional methods. However, this problem can be addressed using frequent and synoptic satellite observations and the methods proposed in this study.

## Conclusions

The first comprehensive estimate of the water storage in large lakes and reservoirs in the YRB is provided in this study. This information is notably difficult to characterize using traditional methods. A 15-year EDR of the water storages in large lakes and reservoirs in the YRB has been established, from which significant short-term changes and inter-annual variabilities have been revealed. Prominent increases were observed for the total water storages in large reservoirs, and this trend was primarily associated with the TGD impoundment and the water volume increase in the TGR. By contrast, the total water storage in large lakes exhibited a decreasing trend in the past 15 years, and the determination of the exact reason requires additional analysis.

This study demonstrated the possibility of optical remote sensing data in monitoring the water storages of large water bodies across a large area. The EDR provided here offers a critical reference for future monitoring and regulation of the water resources in the YRB and China.

## Additional Information

**How to cite this article**: Cai, X. *et al*. Remote Sensing of the Water Storage Dynamics of Large Lakes and Reservoirs in the Yangtze River Basin from 2000 to 2014. *Sci. Rep.* **6**, 36405; doi: 10.1038/srep36405 (2016).

**Publisher’s note:** Springer Nature remains neutral with regard to jurisdictional claims in published maps and institutional affiliations.

## Supplementary Material

Supplementary Information

## Figures and Tables

**Table 1:** Annual mean surface water storages of large lakes and reservoirs in the YRB (km^3^).

Year	Lake Storage	Reservoir Storage	Total Surface Water Storage
2000	30.25	69.57	99.82
2001	28.54	74.09	102.63
2002	29.51	69.14	98.65
2003	29.45	78.28	107.73
2004	26.46	76.24	102.69
2005	29.87	85.09	114.96
2006	28.05	89.70	117.76
2007	26.64	88.79	115.42
2008	26.51	92.76	119.27
2009	26.29	96.77	123.05
2010	30.37	98.93	129.31
2011	25.29	97.90	123.19
2012	29.27	97.88	127.15
2013	26.06	92.96	119.02
2014	27.24	93.83	121.08
mean	27.97	86.89	114.87
