# Supplementary material for: Remote Sensing of the Water Storage Dynamics of Large Lakes and Reservoirs in the Yangtze River Basin from 2000 to 2014
Source: Sci Rep. 2016 Nov 4;6:36405. doi: 10.1038/srep36405 (PMC5095655; doi:10.1038/srep36405)
Supplement: Supplementary Information [file srep36405-s1.docx]

**Remote Sensing of Water Storage Dynamics of Large Lakes and Reservoirs in the Yangtze River Basin from 2000 to 2014**

Xiaobin Cai^a,b^, Lian Feng^c*^, Xuejiao Hou^c^, Xiaoling Chen^c^

^a^ Institute of Geodesy and Geophysics, Chinese Academy of Sciences, Wuhan, 430077, China

^b^ Key Laboratory of Monitoring and Estimate for Environment and Disaster of Hubei, Wuhan, 430077, China

^c^ State Key Laboratory of Information Engineering in Surveying, Mapping and Remote Sensing, Wuhan University, Wuhan 430079, China

*Corresponding to Lian Feng, Email: lian.feng@whu.edu.cn, Tel.: +86-13627131508, State Key Laboratory of Information Engineering in Surveying, Mapping and Remote Sensing, Wuhan University, Wuhan 430079, China


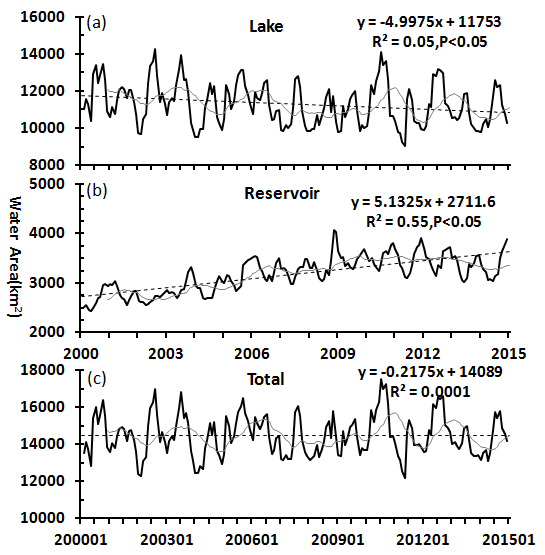


Figure S1. MODIS-derived, long-term monthly mean inundation areas of large lakes (a) and reservoirs (b) in the YRB and their summation (c) from 2000 to 2014.


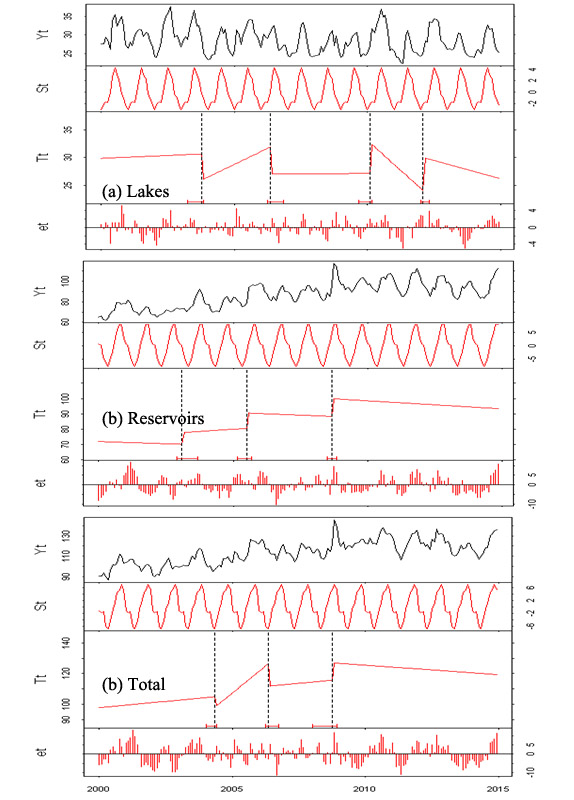


Figure S2. The monthly mean water storages (Yt) from 2000 to 2014, their seasonal (St) and long-term trends (Tt), and the remaining components decomposed using BFAST. The results for lakes, reservoirs and total surface waters are illustrated in (a), (b) and (c), respectively.


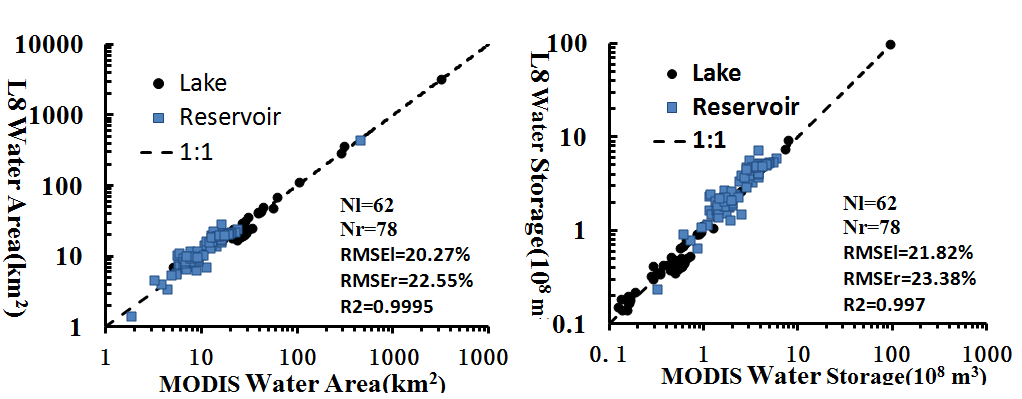


Figure S3. Comparisons between MODIS (250 m)- and Landsat-8 OLI (30 m)-delineated inundation areas (a) and the corresponding water storages (b).


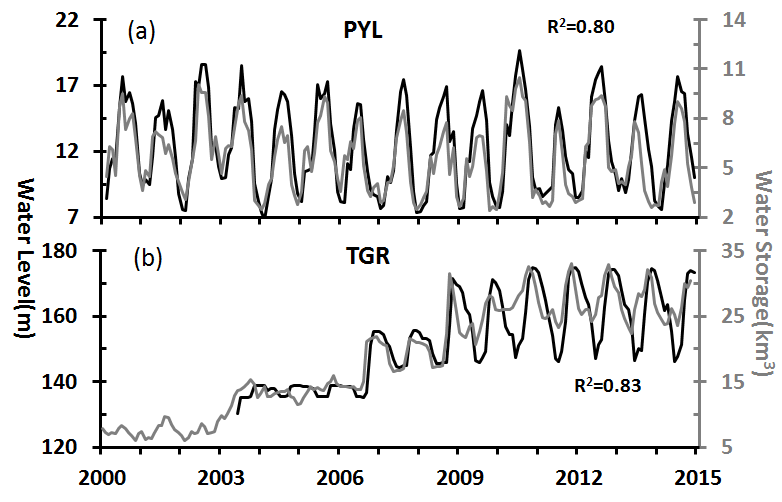


Figure S4. Time series of estimated storage and the concurrent water level in Poyang Lake (a) and Three Gorge Reservoir (b).


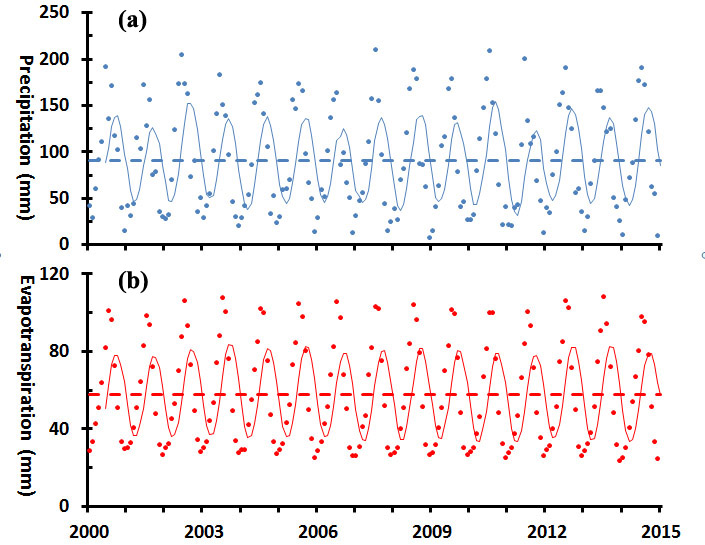


Figure S5. Long-term precipitation (a) and evapotranspiration (b) in the YBR between 2000 and 2014.
